# Prenatal Environmental Exposure to Persistent Organic Pollutants and Indices of Overweight and Cardiovascular Risk in Dutch Adolescents

**DOI:** 10.3390/nu14112269

**Published:** 2022-05-28

**Authors:** Sietske A. Berghuis, Arend F. Bos, Pieter J. J. Sauer, Gianni Bocca

**Affiliations:** 1 Division of Neonatology, Department of Pediatrics, Beatrix Children’s Hospital, University Medical Center Groningen, University of Groningen, Hanzeplein 1, P.O. Box 30.001, 9713 GZ Groningen, The Netherlands; a.f.bos@umcg.nl; 2 Division of Endocrinology, Department of Pediatrics, Beatrix Children’s Hospital, University Medical Center Groningen, University of Groningen, Hanzeplein 1, P.O. Box 30.001, 9713 GZ Groningen, The Netherlands; g.bocca@umcg.nl

**Keywords:** prenatal exposure, longitudinal study, persistent organic pollutant, endocrine disruptor, body mass index, overweight, lipid hormone profile, glucose metabolism, cardiovascular risk, adolescent

## Abstract

Persistent organic pollutants (POPs) may have obesogenic effects. Knowledge about the effects of prenatal exposure to POPs on anthropometric measurements and metabolic parameters into adolescence is limited. Therefore, the aim of the current study was to determine whether prenatal environmental exposure to several POPs is associated with indices of overweight and cardiovascular risk in 13–15-year-old children. In this Dutch observational cohort study, 194 mother–infant pairs were included (1998–2002). Maternal pregnancy serum levels of PCBs, OH-PCBs, PBDEs, and other POPs were measured. At follow-up (2014–2016), levels of cholesterol, HDL-C, LDL-C, triglycerides, fasting insulin, fasting glucose, leptin, and adiponectin were measured in their children. The children’s height, weight, waist circumference, hip circumference, and blood pressure were measured. In total, 101 adolescents (14.4 ± 0.8 years; 53.7% of invited) participated of which 55 were boys. Mean BMI was 19.1 ± 3.6 kg/m^2^ and mean BMI z-score 0.13 ± 1.14. Higher prenatal levels of PCBs were associated with lower levels of HDL-C and adiponectin in boys and higher levels of PBDEs with higher triglycerides in girls. We found significant differences by sex in the associations with OH-PCBs, with lower HDL-C and adiponectin, higher LDL-C/HDL-C ratio, fasting glucose, HOMA2-IR, height, and weight for boys. Our study indicates that higher prenatal exposure to PCBs, OH-PCBs, and PBDEs was associated with adolescent levels of some metabolic cardiovascular risk markers and hormones associated with the development of obesity and cardiovascular disease.

## 1. Introduction

There is growing evidence that persistent organic pollutants (POPs) can increase the risk of obesity and overweight during childhood [1]. Due to their extensive use and resistance to biological and chemical degradation, POPs can persist in the environment for long periods after production and their use has been banned by law. Humans are exposed to environmental chemicals via food, drinking water, and air. POPs include polychlorinated biphenyls (PCBs), polybrominated diphenyl ethers (PBDEs), dichloroethene (DDE, a degradation product of the insecticide dichlorodiphenyltrichloroethane), the wood protective agent pentachlorophenol (PCP), hexabroomcyclododecane (HBCDD), and others. PCBs were produced between 1929 and 1985 for application in a variety of products, including coolants in heat-transfer systems, lubricants, and inks [2]. PCBs are metabolized to hydroxy-PCBs (OH-PCBs) in the liver. Both PCBs and OH-PCBs can be transferred from the mother to the fetus during pregnancy via the placenta [3]. As OH-PCBs can be transferred in a higher ratio compared to PCBs, there is a potential for greater OH-PCB toxicity for the fetus [3]. The prenatal period is a sensitive period during which essential developmental processes are initiated, and disruption of these processes may affect long-term outcomes in later life [4,5]. 

Several studies reported that maternal exposure to POPs can exert obesogenic effects in children [6,7]. A possible mechanism is the “metabolic disruptor” hypothesis described by Heindel et al. [8]. Another mechanism suggested is that adipose tissue might play a role as a modulator and target of the toxicity of POPs, as thoroughly described by La Merril et al. [9]. Another suggested mechanism is interference with epigenetic changes, such as DNA methylation [10].

Regarding the effects of prenatal exposure to POPs on anthropometric measurements in childhood, Stratakis et al. reported, after extensive review of data from several birth cohorts, that prenatal exposures to DDE and HCB were associated with increased BMI in children. For PCBs and PBDEs, there was no conclusive evidence that prenatal exposure to these chemicals was associated with obesity development in childhood [11]. Higher PCB exposure was associated with increasing weight in girls [12], whereas other studies reported reduced weight [13] or no associations with anthropometric measurements [14,15]. Regarding prenatal DDE exposure, higher exposure was associated with an increased BMI [15,16] and with increased height for boys in puberty [12]. We did not find studies reporting on the effects of prenatal OH-PCB and anthropometric measurements during the postnatal period or studies on the effects of prenatal PBDE exposure on anthropometric measurements in adolescence. 

Parameters of the metabolic profile include the lipid profile (including cholesterol, HDL-C, LDL-C, and triglycerides) and parameters for glucose metabolism (including insulin and glucose). Increased LDL-C and decreased HDL-C are potential risk factors for cardiovascular disease. Based on a systematic review and meta-analysis, Li et al. concluded that higher adiponectin levels are associated with a lower risk of type 2 diabetes [17]. López-Jaramillo described aspects of the role of leptin and adiponectin in metabolic syndrome and diabetes [18]. Leptin was found to be an important link between obesity and cardiovascular risk, with paradoxically increased leptin levels in obese individuals. Adiponectin is an adipocyte-derived hormone and adiponectin levels are lower in more obese individuals.

Regarding the effects of prenatal POP exposure and lipid profile and energy metabolism in adolescence, studies are scarce. A recent study in Spanish adolescents reported that higher prenatal levels of PCBs were associated with higher LDL-C in adolescents and higher total levels of cholesterol in girls [19]. Regarding the effects on glucose metabolism, higher prenatal PCB exposure was associated with higher non-fasting insulin in 5-year-old girls, whereas no associations were found for boys or with leptin levels [20]. A Dutch study reported on the effect of prenatal dioxin levels on energy metabolism in adolescence and showed that prenatal dioxin (polychlorinated dibenzo-p-dioxins and polychlorinated dibenzofurans; prenatal levels of PCBs not included) exposure was positively correlated to the glucose:insulin ratio and negatively correlated to the fasting insulin concentration in 14–19-year-old study participants [21].

Knowledge is limited about the effects of prenatal exposure to POPs, particularly regarding OH-PCBs and PBDEs, on metabolic parameters and anthropometric measurements in adolescence. The objective of our study was to determine whether prenatal background exposure to POPs is associated with anthropometric, hormonal, and metabolic indices of overweight and other factors increasing cardiovascular risk in adolescence. We hypothesized that higher prenatal POP exposure is associated with anthropometric measurements and levels of hormones and metabolic cardiovascular markers that lead to a higher cardiovascular risk.

## 2. Materials and Methods

### 2.1. Study Population 

This prospective, longitudinal cohort study is part of the Development at Adolescence and Chemical Exposure (DACE) study, in which we followed up two Dutch cohorts. Between 1998 and 2000, 104 mother–infant pairs were included in the first cohort: the Risk of Endocrine Contaminants on human health (RENCO) study [3]. Between 2001 and 2002, 90 mother–infant pairs were included in the second cohort: the Groningen infant COMPARE (Comparison of Exposure-Effect Pathways to Improve the Assessment of Human Health Risks of Complex Environmental Mixtures of Organohalogens) study, also known as the GIC study [22]. Children of both cohorts were invited for the current study. The children were all singletons and born at term (37–42 weeks’ gestation) without congenital anomalies or diseases. Their mothers were of Western European origin and had no serious illnesses or complications during pregnancy or delivery. All adolescents and their parents provided their written informed consent before participation in the follow-up program. The follow-up and the original study were approved by the University Medical Center Groningen Medical Ethics Committee (2014/029).

### 2.2. Assessment of Prenatal Levels of POPs

Maternal blood samples were taken during the second and/or third trimester of pregnancy. Detailed descriptions of the analyses have been published previously [3,22]. Levels of PCB-153, 4-OH-PCB-107, 4-OH-PCB-146, and 4-OH-PCB-187 were measured in both cohorts. In the RENCO cohort, also nine other PCBs (105; 118; 138; 146; 156; 170; 180; 183; 187) and three other OH-PCBs (3-OH-PCB-153; 3′-OH-PCB-138; 4′-OH-PCB-172) were measured and the sum of all (OH-)PCBs was calculated. The following POPs were also measured in the GIC cohort: 2,2′-bis-(4 chlorophenyl)-1,1′-dichloroethene (p,p’-DDE), pentachlorophenol (PCP), five different 2,2′,4,4′-tetrabromodiphenyl ethers (BDEs) and hexabroomcyclodo-decane (HBCDD). PCBs and OH-PCBs were numbered respectively according to Ballschmiter et al. and to Letcher et al. [23,24]. 

### 2.3. Measurement of Anthropometric Measurements at Adolescence

At adolescence, we measured height, weight, waist circumference, hip circumference, and blood pressure in the lying position. The measurements were performed with children wearing only their underwear. All measurements were performed at the clinic by a trained physician. Weight was determined to the nearest 0.1 kg using a standard electronic calibrated scale for all measurements. Height was determined to the nearest 0.1 cm using the same wall-mounted Ulmer stadiometer (Busse Design + Engineering, Elchingen, Germany) for all measurements. The average of both measurements was used for the calculation of the body mass index (BMI), by dividing the weight in kilograms by the height in meters squared. An age-specific and sex-specific BMI z-score was calculated using the Growth Analyzer version 3 (available at www.growthanalyser.org (accessed on 15 Dec. 2016)), which contains data from the Fourth Dutch Growth Study obtained in 1996 and 1997. The circumferences of the waist and hips were measured using the same Hoechstmass measuring tape (Hoechstmass Balzer GmbH, Sulzbach, Germany). As an indicator of abdominal fat, waist-to-height ratio (WHtR) was calculated as waist (cm)/height (cm). Blood pressure was measured at the right arm after one minute in the supine position using a Suresigns VS2 automatic arm blood-pressure cuff (Philips Medical Systems, Andover, MA, USA). All measurements were taken in duplicate, and the average of both measurements was used for statistical analyses. 

### 2.4. Assessment of Hormones and Cardiovascular Markers at Adolescence

Blood samples were taken by venipuncture at 8:30 a.m. after an overnight fast, except for three samples which were taken around 10.30 a.m. One BD Vacutainer K2E (EDTA) 18.0 mg of 10 mL was used for obtaining plasma samples and a BD Vacutainer SST II Advance of 5 mL for obtaining serum samples (Becton Dickinson, Franklin Lakes, NJ, USA). A BD Vacutainer Sodium Fluoride of 2 mL was used for obtaining blood samples for measurement of fasting glucose levels (Becton Dickinson, Franklin Lakes, NJ, USA). Glucose levels were measured on the same day the venipuncture was taken. The samples were centrifuged (4 °C; 211 r (mm); 2347 n (rpm); 1300 RZB (g); t (min) = 11.20), and the plasma and serum were transferred to plastic tubes and stored at −20 °C until analysis. Serum cholesterol levels were measured by enzymatic and colorimetric method Roche/Hitachi Cobas C (Roche Diagnostics GmbH, Mannheim, Germany) with a measuring range of 0.1–20.7 mmol/L, and a lower detection limit of 0.1 mmol/L. Serum HDL-C and LDL-C cholesterol levels were measured using homogeneous enzymatic colorimetric test Roche/Hitachi Cobas C (Roche Diagnostics GmbH, Mannheim, Germany), with measuring ranges of 0.08–3.88 mmol/L and 0.10–14.2 mmol/L, respectively, and an LOD of 0.08 mmol/L and 0.10 mmol/L, respectively. Triglycerides were measured using enzymatic colorimetric test Roche/Hitachi Cobas C (Roche Diagnostics GmbH, Mannheim, Germany) with a measuring range of 0.1–10.0 mmol/L and a LOD of 0.1 mmol/L. Serum fasting insulin levels were measured using chemiluminescent microparticle immunoassay ‘Alinity i Insulin essay’ (Abbott GmbH & Co. KG, Wiesbaden, Germany) with a measuring interval of 1.6 to 300.0 μU/mL and a limit of detection (LOD) of 0.4 μU/mL. Plasma fasting glucose levels were measured using enzymatic hexokinase method Roche/Hitachi Cobas C (Roche Diagnostics GmbH, Mannheim, Germany) with a measuring range of 0.11–41.6 mmol/L and a lower detection limit of 0.11 mmol/L. Plasma leptin and adiponectin levels were measured using quantitative sandwich enzyme immunoassay Quantikine ELISA immunoassay (R&D Systems, Inc, Minneapolis, MN, USA) with typically minimum detectable doses of 7.8 pg/mL and 0.246 ng/mL, respectively. Homeostatic model assessment of insulin resistance (HOMA2-IR) was performed using the HOMA 2 Calculator © Oxford University 2004 (available at www.dtu.ox.ac.uk/homacalculator/download.php (accessed on 25 February 2022)), inserting values of insulin levels measured in serum and values of glucose levels as measured in plasma. 

### 2.5. Statistical Analyses

The levels of the POPs were log10-transformed to adjust for skewed distribution. Multivariable linear regression analyses were performed to assess the association between prenatal compound levels and anthropometric outcomes, hormone levels, and cardiovascular markers. As they were considered confounders, age at examination (in months) and sex were included in all multivariable models. Maternal pre-pregnancy body mass index (BMI), maternal age at delivery (in years), maternal smoking during pregnancy (yes/no), and maternal education (<14/≥14 years’ school education) were considered as confounders and were included in the multivariable models if the factor was significantly associated with the outcome measure in a univariate linear regression model. Multivariable linear regression analyses were performed to assess the association between 34 POP variables and 18 outcome measures for the complete cohort and also for the girls and the boys separately (Figures S1–S3). To explore the possible interaction between POP level and sex, interaction terms were computed and added to the multivariable linear regression models (Figure S4). A *p*-value below 0.05 was considered statistically significant, and a value between 0.05 and 0.10 was considered a trend towards significance. Statistical Package for the Social Sciences, version 23 (SPSS) was used.

## 3. Results

### 3.1. Study Group

Characteristics of the study group are presented in Table 1. Of the 188 children invited, 101 (53.7 %) participated. Forty-four (23.4%) declined the invitation and forty-three (22.9%) did not respond after a reminder. All children were 13–15 years old, except one girl who turned 16 the day prior to the follow-up visit. The mean age of the mother at delivery was 32 years and the mean pre-pregnancy body mass index (BMI) 24.5 kg/m^2^. The POP levels of the mother–infant pairs included in this study were reported previously [25]. The POP levels did not differ between the included and excluded children, except for PBDE-154, which was lower in included children (0.50 ± 0.24 versus 0.84 ± 0.73 ng/g lipids; t = −2.573; *p* = 0.028). The median level and interquartile range for PCB-153 and sum of the 10 PCBs were 76.7 (52.0–104.6) and 319.0 (244.2–401.1) ng/g lipids, respectively. The median sum of the six OH-PCBs was 377.5 (276.5–540.5) pg/g fresh weight, and for DDE it was 85.9 (64.2–127.9) ng/g lipids.

### 3.2. Maternal Characteristics and Outcomes in Offspring at Adolescence 

Maternal pre-pregnancy BMI was positively associated with leptin (beta = 0.321; *p* = 0.003), waist circumference (beta = 0.215; *p* = 0.033), hip circumference (beta = 0.263; *p =* 0.009), waist/height ratio (beta = 0.286; *p* = 0.004), and BMI z-score in offspring at adolescence (beta = 0.306; *p* = 0.002). Maternal smoking during pregnancy (yes versus no) was associated with higher weight at adolescence (beta = 0.217; *p* = 0.029) and higher systolic blood pressure (beta = 0.212; *p* = 0.033). Higher education level of the gravida (≥14 years’ versus <14 years’ school education) was associated with lower triglycerides (beta = −0.233; *p =* 0.028) and higher HDL-C in offspring at adolescence (beta = 0.204; *p* = 0.045). Maternal parity history (multiparous versus nulliparous) was associated with lower triglycerides in offspring at adolescence (beta = −0.229; *p =* 0.024). Maternal age at delivery was not significantly associated with any of the reported outcomes in offspring at adolescence.

### 3.3. Anthropometric Measurements and Blood Pressure

Anthropometric measurements and blood pressure are presented in Table 1. The mean height was 169.9 ± 8.3 cm, the mean weight was 58.1 ± 13.1 kg, the mean BMI was 19.1 ± 3.6 kg/m^2^, and the mean BMI z-score was 0.13 ± 1.14. Of the 55 boys included in the study, 4 (7%) were overweight, as defined by a BMI z-score > 1.1 until 2.3, and 4 (7%) had obesity, as defined by a BMI z-score > 2.3. Of the 46 girls included in the study, 11 (24%) were overweight, and there were no girls with a BMI z-score > 2.3. The mean circumferences of the waist and hips were 70.1 ± 8.2 and 81.4 ± 8.6 cm, respectively. The mean systolic and diastolic blood pressures were 116.6 ± 9.0 and 59.2 ± 6.8 mmHg, respectively.

### 3.4. Hormones and Metabolic Cardiovascular Markers

In Table 2, the adolescent levels of the metabolic parameters measured in boys and girls are presented. For four children, no blood samples were available due to refusal of venipuncture. Due to logistic reasons, for nine children no plasma samples were available. For three children, the levels of glucose were not were measured due to the absence of a fasting period before venipuncture. The other metabolic outcomes measured in serum and plasma of these three children were included in the statistical analyses. Levels of leptin were negatively correlated with HDL-C and positively with cholesterol, LDL-C, triglycerides, and insulin (all *p* < 0.05; Spearman correlation test). Adiponectin was negatively correlated with insulin and positively with HDL-C. Leptin and adiponectin were negatively correlated. BMI z-score was positively correlated with leptin, LDL-C, and insulin and negatively with adiponectin, HDL-C, and fasting glucose (all *p* < 0.05; Spearman correlation test).

### 3.5. Prenatal PCB Levels and Indices for Overweight and Cardiovascular Risk in Adolescence

**Figure 1:**
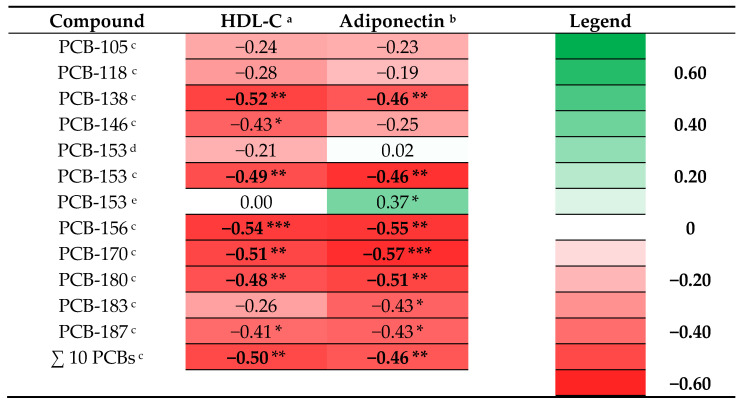
Heat map of adjusted beta coefficients obtained from a selection of the performed linear regression analyses between prenatal POPs and outcome measurements: log10 transformed prenatal PCB levels and HDL-C and adiponectin in adolescent boys. * *p* ≤ 0.10; ** *p* ≤ 0.05; *** *p* ≤ 0.01. (HDL-C: High-density lipoprotein cholesterol; ^a^ adjusted for age at examination and maternal education; ^b^ adjusted for age at examination; ^c^ RENCO cohort, respectively *n* = 26 and *n* = 20; ^d^ total cohort, respectively *n* = 54 and *n* = 48; ^e^ GIC cohort, *n* = 28).

Heat maps of the beta coefficients of multivariable linear regression analyses including log10-transformed prenatal PCB levels and outcome measurements, adjusted for age at examination and sex and maternal characteristics for the complete cohort are presented in Supplementary Figure S1 and for both sexes separately in Supplementary Figures S2 and S3. A pattern of associations between prenatal PCB levels and adolescent levels of HDL-C and adiponectin in boys was observed and highlighted in Figure 1 (Figure 1). Prenatal levels of 5 out of the 10 PCBs and the sum of all 10 PCBs measured in the RENCO cohort were significantly associated with lower levels of HDL-C in boys, and for 2 other PCBs a similar marginally significant association was found (Figure 1). Prenatal levels of 5 out of the 10 PCBs and the sum of all 10 PCBs measured in the RENCO cohort were significantly associated with lower levels of adiponectin in boys, and for 2 other PCBs a similar marginally significant association was found (Figure 1). In Supplementary Figure S4 we present the beta coefficients for the associations between the interaction terms Log10-POP*sex (male = 1; female = 0) and outcome measurements (Figure S4). The effect of log10-PCB-153 was significantly different according to sex, with higher HOMA2-IR and height for boys (Figure S4).

### 3.6. Prenatal PBDE Levels and Indices for Overweight and Cardiovascular Risk in Adolescence

**Figure 2:**
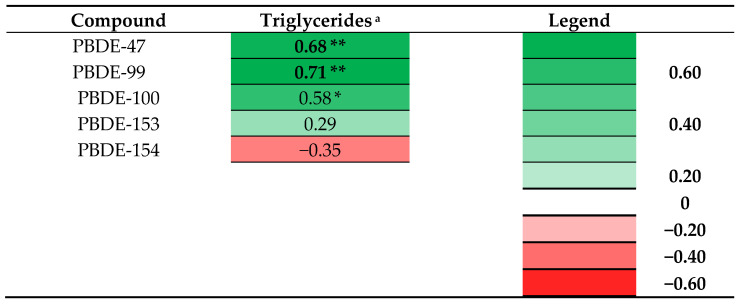
Heat map of adjusted beta coefficients obtained from a selection of the performed linear regression analyses between prenatal POPs and outcome measurements: log10 transformed prenatal PBDE levels and triglycerides in adolescent girls. * *p* ≤ 0.10; ** *p* ≤ 0.05. (^a^ adjusted for age at examination, maternal education, and parity; *n* = 13).

Heat maps of the adjusted beta coefficients of multivariable linear regression analyses including log10-transformed prenatal PBDE levels and outcome measurements are presented in Supplementary Figures S1–S3. With regard to PBDE levels, a pattern of associations between prenatal PBDE levels and adolescent levels of triglyceride in girls was observed and highlighted in Figure 2. Prenatal levels of two PBDEs were significantly associated with higher triglycerides in girls and for one PBDE and the sum of all five PBDEs similar marginally significant associations were found. With regard to outcomes in boys, only higher PBDE-154 was associated with higher systolic blood pressure at adolescence and higher PBDE-47 was marginally significantly associated with higher levels of adiponectin (Figure S2).

### 3.7. Prenatal OH-PCB, DDE, PCP, HBCDD, and Outcomes in Adolescence

With regard to prenatal levels of OH-PCBs, some significant or marginally significant associations were found with anthropometric measurements or metabolic parameters (Figures S1–S3). In Supplementary Figure S4 we present the beta coefficients for the association between the interaction terms Log10-POP*sex (male = 1; female = 0) and outcome measurements (Figure S4). With regard to interaction terms on prenatal OH-PCBs*sex and HDL, the effect of OH-PCB-146 was significantly different according to sex, with lower HDL values for boys. The interaction terms of three other OH-PCBs were also negative, although marginally significant (Figure S4). Significant positive interactions, indicating higher values in boys, were found for three OH-PCBs and the LDL-C/HDL-C ratio, and a marginally significant interaction was found for one OH-PCB compound (Figure S4). Further negative interactions, indicating lower levels in boys, were observed for the association between three OH-PCBs (OH-PCB-107, OH-PCB-172 and OH-PCB-187) and adiponectin, and for OH-PCB-146 a similar marginally significant interaction was found (Figure S4). For the compounds OH-PCB-107 and OH-PCB-187, the effects were significantly different according to sex, with higher fasting glucose, HOMA2-IR, height, and weight for boys (the latter for OH-PCB-107 was marginally significant; Figure S4).

With regard to prenatal levels of DDE, prenatal levels of DDE were marginally significant negatively associated with waist circumference, waist-to-height ratio, and BMI z-score in boys and with height in girls. Prenatal levels of DDE were not significantly associated with indices for overweight or cardiovascular risk (including lipid profile), parameters of glucose metabolism, or the hormones leptin or adiponectin. With regard to prenatal levels of PCP, only some significant or marginally significant associations were found. Higher prenatal PCP levels were associated with higher BMI and weight in the complete cohort (girls and boys), with significantly higher glucose and lower insulin levels in girls, and marginally significantly higher BMI z-scores were found in boys (Figures S1–S3). Prenatal levels of HBCDD were not significantly associated with any of the outcome measurements.

## 4. Discussion

Our explorative study indicates that higher prenatal exposure to PCBs, OH-PCBs, and PBDEs was associated with adolescent levels of some cardiovascular risk markers and hormones that are associated with the development of obesity and cardiovascular disease. The first important finding is that higher prenatal PCB levels are associated with lower levels of HDL-C and adiponectin in boys. The second important finding is that higher prenatal PBDE levels are associated with higher triglycerides in girls at adolescence. A third finding is that the effects of OH-PCBs were found to be significantly different according to sex, with lower HDL-C, higher LDL-C/HDL-C ratio, and lower adiponectin for boys.

In boys, prenatal levels of PCBs are significantly associated with lower levels of HDL-C and lower levels of adiponectin. This seems in contrast to the findings of a Spanish cohort including 219 fourteen-year-old children, where no association was found between prenatal PCB levels and levels of HDL-C [19]. In a cohort of 6–7-year-old children, no associations were found between maternal PCB levels and levels of HDL-C, either [26]. The latter study reported that maternal PCB-138 was associated with lower childhood levels of triglycerides, LDL-C, and total lipids. In adults, Aminian et al. reported a positive relation between adult plasma PCB levels and serum triglyceride levels [27]. With regard to the association between PCB levels and adiponectin, no data is available on prenatal PCB levels and adiponectin levels at adolescence. With regard to adult levels of PCBs, Lim et al. reported that PCB-28 and PCB-153 were inversely associated with adiponectin in women and that in the high BMI group within their study, PCB-153 showed significant negative associations with adiponectin levels [28]. The findings in our study and in the study by Lim et al. both suggest that PCB exposure might be associated with lower adiponectin levels. Based on outcomes in the mentioned birth cohorts and underlined by the results in our study, there seem to be sex-specific associations between exposure to POPs and metabolic outcomes. 

The second important finding is that higher prenatal levels of PBDEs were found to be associated with higher triglycerides in girls. Data suggest that elevated triglyceride levels are an independent causal risk factor for atherosclerotic cardiovascular disease [29]. To the best of our knowledge, no other studies have been performed on the effects of prenatal exposure to PBDEs and lipid hormone profile at adolescence and, therefore, there are no studies to compare our results with. In a Canadian cohort including 6–7-year-old children, Boutot et al. reported that higher in utero BDE-99 exposure was associated with lower childhood levels of triglycerides and non-significantly with higher HDL-C and lower total lipids [26]. An explanation for the differences between the directions of the associations might be the differences in the age of children included in the study. More research is required to investigate the association between prenatal PBDE exposure and lipid profile at adolescence and adulthood.

A third finding is that the effects of OH-PCBs were found to be significantly different according to sex, with lower HDL-C, higher LDL-C/HDL-C ratio, and lower adiponectin for boys. Further significant interactions were found for the compounds OH-PCB-107 and OH-PCB-187, with higher fasting glucose, higher HOMA2-IR, lower adiponectin, and greater height and weight for boys. The fact that these associations were not found to be significant in the models for both sexes separately might be due to the sample size for the latter analyses. To the best of our knowledge, this is the first study to report on the associations between prenatal OH-PCB exposure and anthropometric measurements, lipid hormone profile, and cardiovascular markers at adolescence and, therefore, there are no studies to compare our results with. The results of our exploratory study suggest that prenatal OH-PCB exposure might exert obesogenic effects, and that these effects can be different according to sex. Larger studies are needed to confirm this finding. A last noteworthy finding is that we did not find a pattern of associations which could suggest that prenatal DDE exposure has an effect on anthropometric measurements or metabolic profile at adolescence. This is in line with three studies reporting the absence of effects on BMI in 5-year-old children on the Faroe Islands [30] and in 5–9-year-old children in Greenland, Poland, and Ukraine [31]. In a study in the US, for children followed until the age of 7 years, no effect on BMI was found [32]. Our findings are in contrast to other studies reporting obesogenic effects of DDE. In children until the age of 7 years, higher prenatal DDE exposure was associated with higher BMI, abdominal obesity, higher diastolic blood pressure [33,34,35], larger waist circumference and WHtR in girls [36], or larger waist circumference in boys [34]. Higher prenatal DDE exposure was associated with an increased BMI in 12-year-old boys, whereas no associations were found in girls [16] and a positive association between maternal DDE exposure and BMI was also found in 20–50-year-old women living in Michigan [15]. Cano-Sancho et al. applied meta-analyses and an integrated systematic review including epidemiological, in vivo and in vitro studies, and concluded that DDE is ‘presumed’ to be obesogenic for humans [37]. The fact that we did not find associations between prenatal DDE levels and indices for overweight or cardiovascular risk might be due to the small sample size or lower exposure levels in our study. Compared to the mentioned study by Karmaus et al., the participants included in our cohort are younger, which might also play a role in the differences [15]. Based on the previously mentioned studies, we speculate that associations between prenatal DDE exposure and BMI might become more evident at later ages.

Several explanations for the obesogenic effects of POPs have been suggested [38,39]. POPs can interfere with hormonally responsive tissue functions via dysregulation of hormone signaling and cell function [38]. A possible mechanism for the obesogenic effects of PCBs is induction of adipocyte differentiation and pro-inflammatory adipokine expression [40]. Arsenescu et al. reported, based on in vitro study, that low concentrations of coplanar “dioxin-like” PCB-77 (3.4 µM) induce adipocyte differentiation and pro-inflammatory adipokines. One of their findings was that adiponectin mRNA expression and concentrations in cell media were decreased by PCB-77. Administering PCB-77 to wild-type mice caused increase in bodyweight gain. These effects were not found in aryl hydrocarbon receptor (AhR)-deficient mice, which suggests that these effects are mediated by the AhR. Arsenescu et al. reported that nonplanar PCB-153 did not induce adipocyte differentiation and pro-inflammatory adipokine expression. The authors suggest that interactions with the AhR, for which PCB-77 possesses affinity but PCB-153 has low affinity, may contribute to these differences in the effects of these PCBs. In obese adults, levels of PCB-138 in adipose tissue were negatively associated with gene-expression levels of adiponectin in visceral adipose tissue [41]. A second suggested mechanism is that endocrine disrupting chemicals can influence epigenetic processes [42]. Stel and Legler concluded, based on a review of literature, that the obesogenic effects of endocrine disrupting chemicals, such as PBDE-47 (PCBs were not included in the review), are mediated by the activation and associated altered methylation of peroxisome proliferator-activated receptor, the master regulator of adipogenesis, or its target genes [42]. With regard to the proposed mechanisms, it is important to mention that there can be differences between ethnic groups [43]. For example, ethnic differences were observed with regard to the effects of the fat-mass- and obesity-associated gene (FTO) on obesity traits [44]. 

A strength of our study is that we assessed both anthropometric and biochemical measurements, including anthropometrics, lipid profile, adipokines, and parameters of glucose metabolism. A second strength is that all measurements at the follow-up at adolescence were performed at the clinic by a trained physician. Other strengths include that almost all children were still living in the northern part of the Netherlands, which minimizes the variability in postnatal exposure levels due to the living environment, and that the results were adjusted for maternal characteristics. A limitation of our study is the possibility of Type 1 errors due to the large number of comparisons. We assessed whether there was a relation between 34 POP values and 18 different outcome measures, resulting in 612 comparisons per group. The results of our explorative study should therefore be interpreted with caution. We base our conclusions on patterns of associations instead of individual *p*-values. However, using a *p*-value of <0.10, our findings showed that 8 out of the 13 (62%) PCB variables were negatively associated with HDL-C in boys, 8 out of the 13 (62%) were negatively associated with adiponectin in boys, and 4 out of 6 (67%) PBDE-variables were positively associated with triglycerides in girls, which is rather unlikely to be explained only by chance. We believe that our analyses were justified as part of a careful evaluation of a rich dataset in hypothesis-driven research [45]. Larger studies are needed to confirm our findings. Whether our findings might have consequences for later life needs to be studied. Future research should focus on the effects of prenatal exposure to POPs on cardiovascular risk factors at adulthood, on sex-specific effects, and on whether epigenetic changes might play a role in the associations between prenatal POP exposure and indices for cardiovascular risk at adolescence.

## 5. Conclusions

In conclusion, higher prenatal Dutch background exposure to PCBs was found to be associated with lower levels of HDL-C and adiponectin in 13–15-year-old boys and higher prenatal exposure to PBDEs with higher triglycerides in girls. We found significant differences by sex on the associations between higher prenatal OH-PCB levels and outcome measures, with lower HDL-C, higher LDL-C/HDL-C ratio, lower adiponectin, higher fasting glucose, higher HOMA2-IR, and greater height and weight for boys. Our findings are in line with our hypothesis that higher prenatal exposure to POPs is associated with changes in levels of hormones and cardiovascular risk markers which indicate an increasing cardiovascular risk. Our finding that prenatal exposure to some POPs is associated with increased levels of cardio-metabolic risk factors at adolescence raises concern regarding the effects of man-made compounds on human health.

## Figures and Tables

**Table 1:** Characteristics of the study group and anthropometric measurements (*n* = 101).

Characteristic	Value	
Sex (boy/girl)	55/46 (54.5/45.5%)	
Age at examination (years)	14.4 ± 0.8	
Gestational age (weeks)	40 (37–42)	
Maternal age at delivery (years)	32.0 ± 3.8	
Maternal pre-pregnancy body mass index (BMI; kg/m^2^) ^a^ (*n* = 98)	24.5 ± 4.0	
Maternal education [<14/≥14 years’ school education]	50/51 (50/51%)	
Maternal smoking during pregnancy [yes/no]	13/88 (13/87%)	
Maternal alcohol consumption during pregnancy [yes/no]	21/80 (21/79%)	
Maternal parity history [nulliparous/multiparous]	36/65 (36/64%)	
	Boys (*n* = 55)	Girls (*n* = 46)
Height (cm)	172.5 ± 9.1	166.8 ± 6.2
Weight (kg)	58.4 ± 15.4	57.7 ± 9.9
Body mass index (BMI; kg/m^2^) ^a^	19.5 ± 3.8	20.7 ± 3.2
BMI z-score ^b^	0.03 ± 1.20	0.24 ± 1.07
BMI-SDS > 1.1 (overweight)	4 (7%)	11 (24%)
BMI-SDS > 2.3 (obese)	4 (7%)	0 (0%)
Waist circumference (cm)	70.8 ± 9.0	69.1 ± 7.2
Hip circumference (cm)	80.0 ± 9.0	83.0 ± 7.9
Waist/height ratio ^c^	0.4 ± 0.0	0.4 ± 0.0
Systolic blood pressure (mmHg) ^d^	119.1 ± 9.1	113.7 ± 8.0
Diastolic blood pressure (mmHg) ^d^	59.0 ± 6.7	59.4 ± 7.0

Data are given as frequencies (n/n), medians (min-max), or means ± SDs; ^a^ BMI calculated as weight/(height in m*height in m); ^b^ based on Growth Analyzer version 3 (http://www.growthanalyser.org/ (accessed on 15 December 2016)); ^c^ waist (cm) divided by height (cm); ^d^ blood pressure was measured in supine position.

**Table 2:** Levels of metabolic parameters measured in serum or plasma after an overnight fast in 13–15-year-old children.

Variable	Boys				Girls			
	*n*	Median	Min	Max	*n*	Median	Min	Max
Insulin (µU/mL serum)	54	8.15	3.50	40.30	43	9.00	3.90	22.50
Fasting glucose (mmol/L plasma)	52	5.20	4.60	6.00	41	5.20	4.00	5.90
Cholesterol (mmol/L serum)	54	3.65	2.34	5.92	43	3.92	2.90	5.73
HDL-C (mmol/L serum)	54	1.43	0.87	2.39	43	1.41	0.91	2.23
LDL-C (mmol/L serum)	54	1.95	0.93	4.10	43	2.22	1.30	3.76
Triglycerides (mmol/L serum)	54	0.72	0.35	1.94	43	0.79	0.36	1.82
Leptin (ng/mL plasma)	48	1.93	0.16	49.06	40	11.48	2.52	62.58
Adiponectin (in ng/mL plasma) ^c^	48	16.80	3.32	32.24	39	12.41	3.35	26.50
LDL-C/HDL-C ratio (mean ± SD)	54	1.53 ± 0.63			43	1.70 ± 0.69		
HOMA2-IR ^d^ (mean ± SD)	52	1.16 ± 0.54			41	1.27 ± 0.51		

HOMA2-IR: homeostasis model assessment of insulin resistance; ^a^ for *n* = 4, the levels of glucose were not measured; ^b^ for *n* = 9, no plasma samples were available; ^c^ for *n* = 1, no exact level of adiponectin was available; ^d^ serum insulin levels and plasma glucose levels were included in HOMA2-calculator.

## Data Availability

Data is available on request.

## Supplementary Materials

The following supporting information can be downloaded at: https://www.mdpi.com/article/10.3390/nu14112269/s1, Figure S1: Heat map of adjusted beta coefficients obtained from linear regression analyses performed between log10 transformed prenatal POP levels and parameters in adolescents; Figure S2: Heat map of adjusted beta coefficients obtained from linear regression analyses performed between log10 transformed prenatal POP levels and parameters in adolescent boys; Figure S3: Heat map of adjusted beta coefficients obtained from linear regression analyses performed between log10 transformed prenatal POP levels and parameters in adolescent girls; Figure S4: POP by sex interaction term significance. Heat map of adjusted beta coefficients for interaction between log10 transformed POP levels and sex, obtained from multivariable linear regression analyses of biochemical and anthropometric parameters in adolescents.
