# Supplementary material for: Prenatal Environmental Exposure to Persistent Organic Pollutants and Indices of Overweight and Cardiovascular Risk in Dutch Adolescents
Source: Nutrients. 2022 May 28;14(11):2269. doi: 10.3390/nu14112269 (PMC9183073; doi:10.3390/nu14112269)
Supplement: Supplementary file 1 [file nutrients-14-02269-s001.zip › nutrients-1710538-supplementary.pdf]

Figure S1. Heat map of adjusted beta coefficients obtained from linear regression analyses performed between log10 transformed prenatal POP levels and parameters in adolescents
(only $p < 0.10$ are shown; glucose, LDL/HDL ratio, height and DBP not sign. associated; HBCDD not sign. associated)

Compound	Chole-sterol	HDL-C	LDL-C	Triglyce rides	Insulin	HOMA2-IR	Adipo-nectin	Leptin	BMI	Weight	Waist Circum-ference	Hip circum-ference	Waist/height ratio	SBP
PCB-105												-0.26		
PCB-118	-0.27			-0.28										
PCB-138	-0.44			-0.38										
PCB-146														
PCB-153														
- RENCO	-0.31	-0.25												
- GIC							0.23							
PCB-156	-0.28	-0.35					-0.31							
PCB-170		-0.34					-0.30							
PCB-180		-0.33					-0.28						0.25	0.25
PCB-183														
PCB-187														
∑ 10 PCBs	-0.26	-0.30												
4-OH-PCB-107														
- RENCO	-0.30		-0.24											
- GIC														0.28
3′-OH-PCB-138	-0.24		-0.25	-0.24	0.27									
4-OH-PCB-146														
- RENCO														
- GIC								-0.24		-0.27	-0.27	-0.29		
3-OH-PCB-153														
4′-OH-PCB-172														
4-OH-PCB-187				0.25										
- RENCO					0.31	0.29					0.23	0.25		0.22
- GIC					-0.32	-0.24		-0.26			-0.29			
∑ 6 OH-PCBs														
BDE-47							0.29		-0.36				-0.33	
BDE-99								-0.32						
BDE-100														
BDE-153				0.35										
BDE-154							0.30							0.30
p.p′-DDE														
PCP									0.26	0.29				

Legend
0.6
0.4
0.2
0
-0.2
-0.4
-0.6

Figure S2. Heat map of adjusted beta coefficients obtained from linear regression analyses performed between log10 transformed prenatal POP levels and parameters in adolescent boys (only $p < 0.10$ are shown; LDL-C, weight, hip circumference and DBP not sign. associated; HBCDD not sig. associated)

Compound	Chole-sterol	HDL-C	LDL/HDL ratio	Triglycerides	Insulin	Glucose	HOMA2-IR	Adipo-nectin	Leptin	BMI z-score	Height	Waist circum-ference	Waist/height ratio	SBP
PCB-105	-0.38				-0.37		-0.39		-0.49					
PCB-118														
PCB-138		-0.52						-0.46						
PCB-146		-0.43												
PCB-153														
- RENCO		-0.49				-0.36		-0.46						
- GIC						0.36		0.37						
PCB-156	-0.36	-0.54				-0.37		-0.55						
PCB-170		-0.51						-0.57						
PCB-180		-0.48	0.36			-0.36		-0.51		0.43				0.37
PCB-183								-0.43						
PCB-187		-0.41				-0.42		-0.43						
∑ 10 PCBs		-0.50						-0.46						
4-OH-PCB-107														
- RENCO														
- GIC														
3′-OH-PCB-138					0.41									
4-OH-PCB-146		-0.25												
- RENCO							0.37	-0.44						
- GIC		-0.37												
3-OH-PCB-153								-0.41						
4′-OH-PCB-172														
4-OH-PCB-187				0.31										
- RENCO					0.38		0.38					0.37	0.37	
- GIC					-0.37							-0.40	-0.41	
∑ 6 OH-PCBs											0.34			
BDE-47								0.47						
BDE-99														
BDE-100														
BDE-153														
BDE-154														0.51
p.p′-DDE										-0.44		-0.36	-0.54	
PCP					-0.34	0.37								

Legend

0.6
0.4
0.2
0
-0.2
-0.4
-0.6

Figure S3. Heat map of adjusted beta coefficients obtained from linear regression analyses performed between log10 transformed prenatal POP levels and parameters in adolescent girls
(only $p < 0.10$ are shown; hip circumference and DBP not sign. associated; HBCDD not sign. associated)

Compound	Chole-sterol	HDL-C	LDL-C	LDL/HDL ratio	Triglyce rides	Insulin	Glucose	HOMA2-IR	Adipo nectin	Leptin	BMI z-score	Height	Weight	Waist Circum-ference	Waist/height ratio	SBP
PCB-105																
PCB-118																
PCB-138	-0.53		-0.33		-0.56											
PCB-146																
PCB-153					-0.31	-0.32		-0.32				-0.31				
- RENCO	-0.38				-0.36											
- GIC						-0.51		-0.53								0.53
PCB-156																
PCB-170																
PCB-180												-0.35				
PCB-183	0.33				0.40											
PCB-187																
∑ 10 PCBs																
4-OH-PCB-107																
- RENCO	-0.40															
- GIC	0.63		0.50													
3′-OH-PCB-138	-0.61		-0.63		-0.40										0.42	
4-OH-PCB-146		0.31														
- RENCO				-0.60												
- GIC		0.58											-0.47			
3-OH-PCB-153												-0.45				
4′-OH-PCB-172			-0.47	-0.58					0.60							
4-OH-PCB-187							-0.31									
- RENCO																
- GIC																
∑ 6 OH-PCBs	-0.49		-0.44													
BDE-47					0.68		-0.53				-0.51	0.46		-0.46	-0.60	
BDE-99					0.71					-0.54						
BDE-100	0.66		0.56		0.58											
BDE-153									-0.63							
BDE-154																
p.p′-DDE												-0.43				
PCP													0.50			

Legend
0.6
0.4
0.2
0
-0.2
-0.4
-0.6

Figure S4. POP by sex interaction term significance. Heat map of adjusted beta coefficients for interaction between log10 transformed POP levels and sex, obtained from multivariable linear regression analyses of biochemical and anthropometric parameters in adolescents

(only $p \leq 0.10$ are shown; $p < 0.05$ in bold; negative values indicate lower values in males; BMI and hip circumference not sign. associated; HBCDD not sign. associated)

Interaction term	Choles-terol	HDL	LDL	LDL/HDL ratio	Triglyce-rides	Insulin	Glucose	HOMA2-IR	Adipo-nectin	Leptin	Height	Weight	Waist circum-ference	Waist/height ratio	SBP	DBP
PCB-105 * male																
PCB-118 * male																
PCB-138 * male		**-2.57**		2.08	1.86			-2.35								
PCB-146 * male																
PCB-153 * male					1.64			**2.45**			**2.49**	1.55				
- RENCO																
- GIC															-2.77	
PCB-156 * male							-1.23									
PCB-170 * male																
PCB-180 * male						2.73										
PCB-183 * male	-1.08				-1.17											
PCB-187 * male																
∑ 10 PCBs * male																
4-OH-PCB-107 * male	-0.94		-0.82				**1.17**	**1.06**	**-0.99**		**1.34**	0.79				
- RENCO																
- GIC	**-2.40**		**-2.26**				1.51							-1.47		
3′-OH-PCB-138 * male		-1.67		**1.93**												
4-OH-PCB-146 * male		**-1.93**														1.60
- RENCO	2.21	-2.78	**2.86**	**3.58**		2.32			-3.17							
- GIC	**-3.08**	-2.86	-2.89													
3-OH-PCB-153 * male		-1.57		1.52												
4′-OH-PCB-172 * male			1.56	**2.03**					**-2.59**							**1.96**
4-OH-PCB-187 * male		-1.79					**2.61**	**2.57**	**-2.09**		**2.47**	**2.12**	1.88			
- RENCO																
- GIC																
∑ 6 OH-PCBs * male	**4.93**		4.60													
BDE-47 * male					-0.89		0.83			0.66				0.66		
BDE-99 * male																
BDE-100 * male	-1.15														**1.08**	
BDE-153 * male									**0.62**							0.58
BDE-154 * male																
p.p′-DDE * male											**2.74**			-2.16	-2.11	
PCP * male							2.58								**-2.93**	

Legend	
	5
	4
	3
	2
	1
	0
	-1
	-2
	-3
	-4
	-5
